# Clinical and Demographic Profile of Cutaneous Melanoma: Pakistani Perspective

**DOI:** 10.29252/wjps.9.3.296

**Published:** 2020-09

**Authors:** Muhammad Saaiq, Saad Siddiqui

**Affiliations:** Department of Plastic Surgery, National Institute of Rehabilitation Medicine (NIRM), Islamabad, Pakistan

**Keywords:** Melanoma, Cutaneous, Skin, Cancer

## Abstract

**BACKGROUND:**

Cutaneous melanomas (CMs) account for only a small proportion of skin cancers, however these are responsible for most skin cancer deaths. There has been a consistently increasing trend in their incidence across the globe.

**METHODS:**

This prospective case series study spanned over a period of three years. All patients with histologically confirmed CMs were included.

**RESULTS:**

There were 31 patients including 28 males and 3 females with the mean age of 58.25±11.33 years. The histological subtypes included 13 cases (41.93%) of nodular melanoma (NM), 11 patients (35.5%) of acral lentiginous melanoma (ALM), 3 cases (9.67%) of superficial spreading melanoma (SSM) and lentigo maligna melanoma (LMM) and one case (3.22%) of desmoplastic melanoma. Two patients (6.45%) presented with stage II, whereas 21 patients had (67.74%) stage III melanoma. There were 8 patients (25.80%) with stage IV. Time interval between onset of the lesion and first presentation to hospital ranged from 6 to 17 weeks with a mean of 12.45±3.2 weeks. The overall median survival for patients with stage III and IV was 8.75 months. The overall survival for stage II at one year was 100%.

**CONCLUSION:**

CMs more frequently affected males aged ≥58 years. Feet, face, trunk, hands and scalp were the affected anatomical body parts in decreasing order of frequency. NM and ALM were the more common histological subtypes. Majority of patients presented late and advanced stages of melanoma. Awareness about the sinister course of the disease will ensure early presentation with better treatment outcome.

## INTRODUCTION

Cutaneous melanomas (CMs) contribute only 4-11% of all cutaneous cancers; however, these account for the overwhelming majority (i.e. ≥75%) of the skin cancer deaths. The incidence trend varies considerably across the globe. The white race of the New Zealand, Australia, United States, Canada and Europe represent the hardest-hit ones of the world. The incidence rate is reported to be 50 per 100,000 individuals. In the United States, melanoma represents the 5^th^ most commonly encountered malignancy among both males and females.^1^^-^^6^

Whereas, CMs plague certain white races of the world more, and they also occur (with less frequency) among the rest of global populations. A consistent escalation in the incidence over the last half century has been reported from all parts of the world.^7^^-^^9^ The current study was undertaken to document the frequency of CMs in our patients, to analyze the clinical and demographic profile of the sufferers and to assess the survival rate. Hence, an evidence-based study regarding this important public health issue seems necessary.

## MATERIALS AND METHODS

This descriptive case series was carried out at National Institute of Rehabilitation Medicine (NIRM) in Islamabad, Pakistan over a period of three years. The study period spanned from October 2016 to September 2019. All patients who were histologically confirmed for CMs were included. Patients not satisfied to participate in the study were excluded. Convenience sampling method was used. Informed consent was taken from all patients. The study proceeded in accordance with the Helsinki’s Declaration-2013 revision. Anonymity of the participants was ensured.

Thorough clinical evaluation of the patients was performed. The diagnosis was confirmed with an initial excision biopsy including 1-2 mm margin of the surrounding normal skin. Incision or saucerization biopsies were performed when the initial excision of the entire lesion was not appropriate due to anatomic or operational constraints. The local extent of melanoma was assessed with computed tomography (CT) scanning or magnetic resonance imaging (MRI). Metastatic workup included sentinel lymph node biopsy (SLNB), serum lactic dehydrogenase (LDH) assay, CT scan of the chest, abdomen, pelvis, and bone, etc. Staging was performed according to the American Joint Committee on Cancer (AJCC) guidelines using the Tumor, Node, Metastasis (TNM) scoring system.^10^

All patients were managed according to the standard oncologic principles for CMs. Any required reconstructive surgical procedure was instituted as dictated by the reconstructive requirements of the individual patients. The demographic profile of the patients, anatomic location of the CMs, histologic subtype, time interval between onset of the lesion and first presentation to hospital, stage at presentation, type of treatment instituted, complications encountered, and survival were all recorded. The data were subjected to statistical analysis using SPSS software (version 11, Chicago, IL, USA). 

## RESULTS

There were 31 patients with 28 males (90.32%) and 3 females (9.67%). The mean age was 58.25±11.33 years with a range of 33-72 years. The histological subtypes included 13 cases (41.93%) of nodular melanoma (NM), 11 patients (35.5%) of acral lentiginous melanoma (ALM), 3 cases (9.67%) of superficial spreading melanoma (SSM) and lentigo maligna melanoma (LMM) and one case (3.22%) of desmoplastic melanoma. Anatomic locations involved foot (n=13, 41.93%), face (n=9, 29%), trunk (n=4, 12.90%), scalp (n=3, 9.67%) and hands (n=2, 6.45%).

**Fig. 1:**
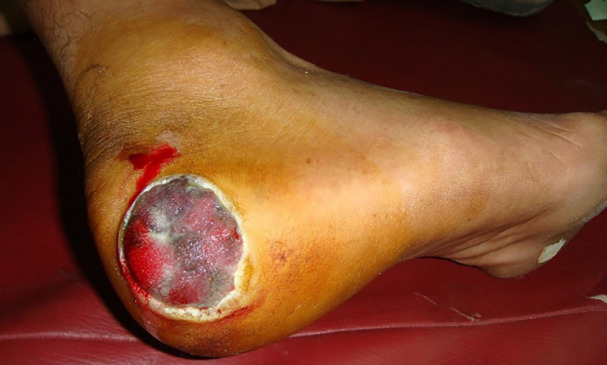
Acral lentiginous melanoma on the right hind foot area of a 59 years old male

Stage distributions at presentation were stage II (n=2, 6.45%), stage III (n=21, 67.74%) and stage IV (n=8, 25.80%). Time interval between onset of the lesion and first presentation to hospital ranged from 6 to 17 weeks, with a mean of 12.45±3.2 weeks. The instituted treatments were surgical excision with curative intent (n=8, 25.80%), regional nodal clearance (n=8, 25.80%), adjuvant radiotherapy (n=6, 19.35%), targeted therapy/immunotherapy (n=16, 51.61%), palliative pain and nutritional management (n=7, 22.58%). The overall median survival for patients with stage III and IV of the disease was 8.75 months. The overall survival for stage II of the disease at one year was 100%. Figures 1 through 8 are clinical photographs of some of the included patients.

**Fig. 2:**
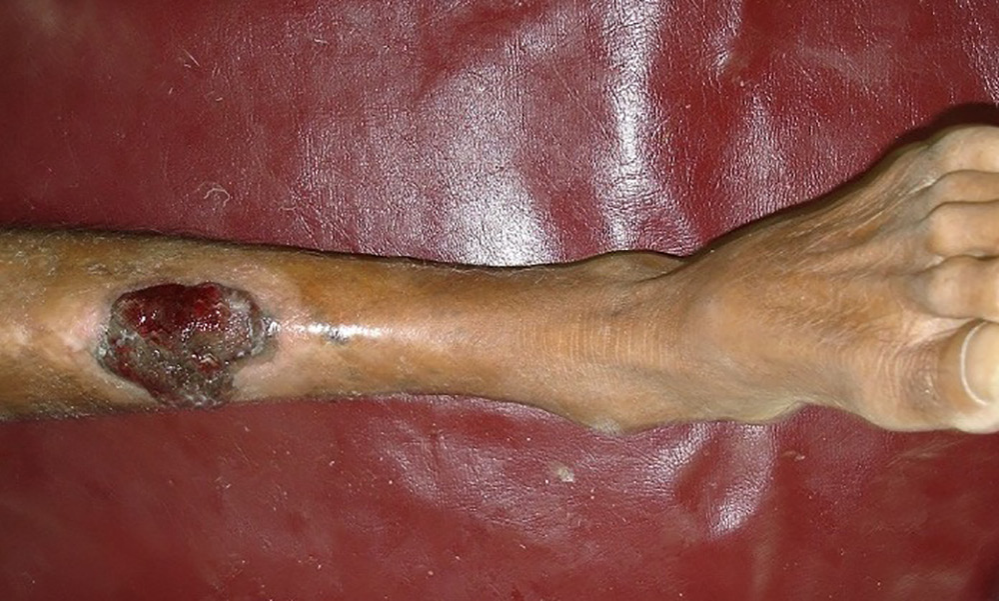
Acral lentiginous melanoma involving the left leg of a 63 years old male

**Fig. 3:**
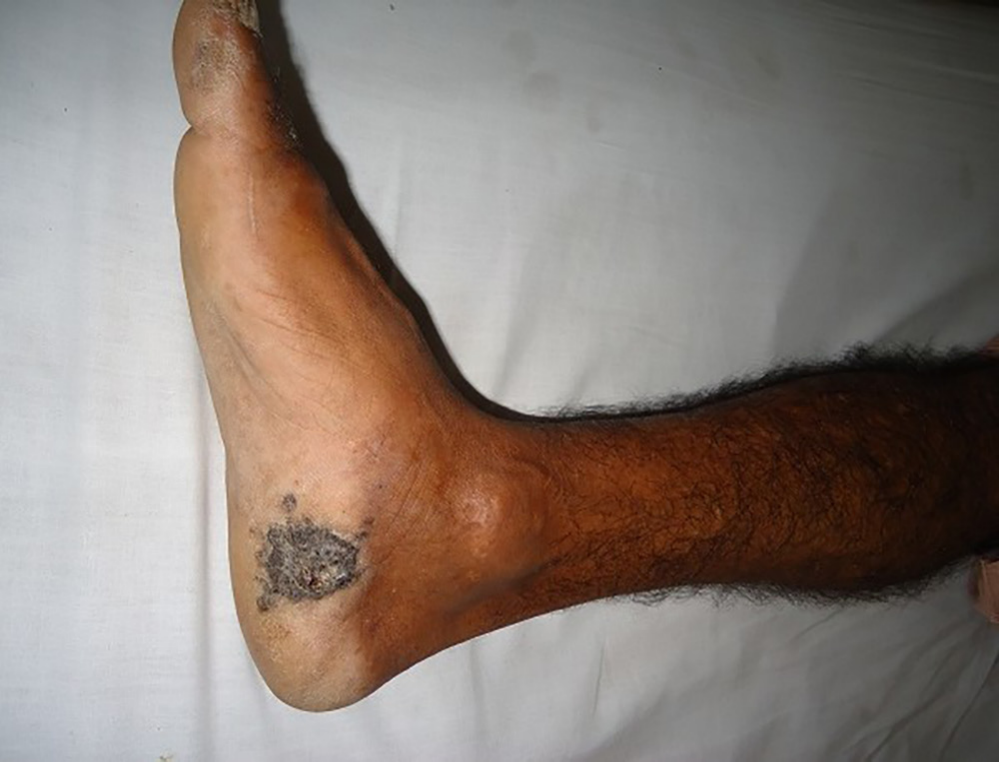
Acral lentiginous melanoma affecting the right foot plantar area of a 61 years old man

**Fig. 4:**
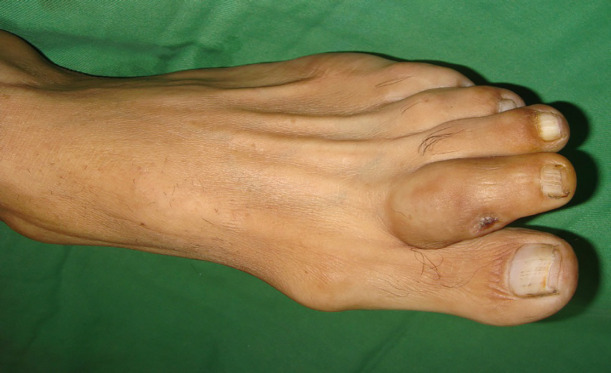
Desmoplastic melanoma involving the second toe of left foot of a 58 years old man

**Fig. 5:**
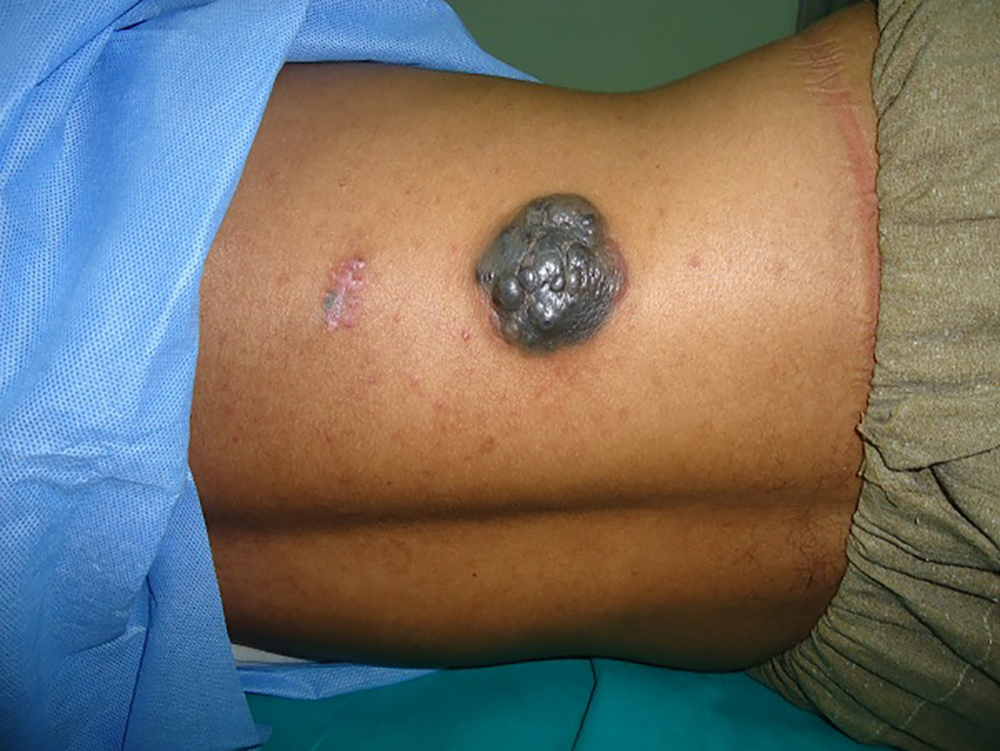
Nodular melanoma on the right side of trunk of a 33 years old man

**Fig. 6:**
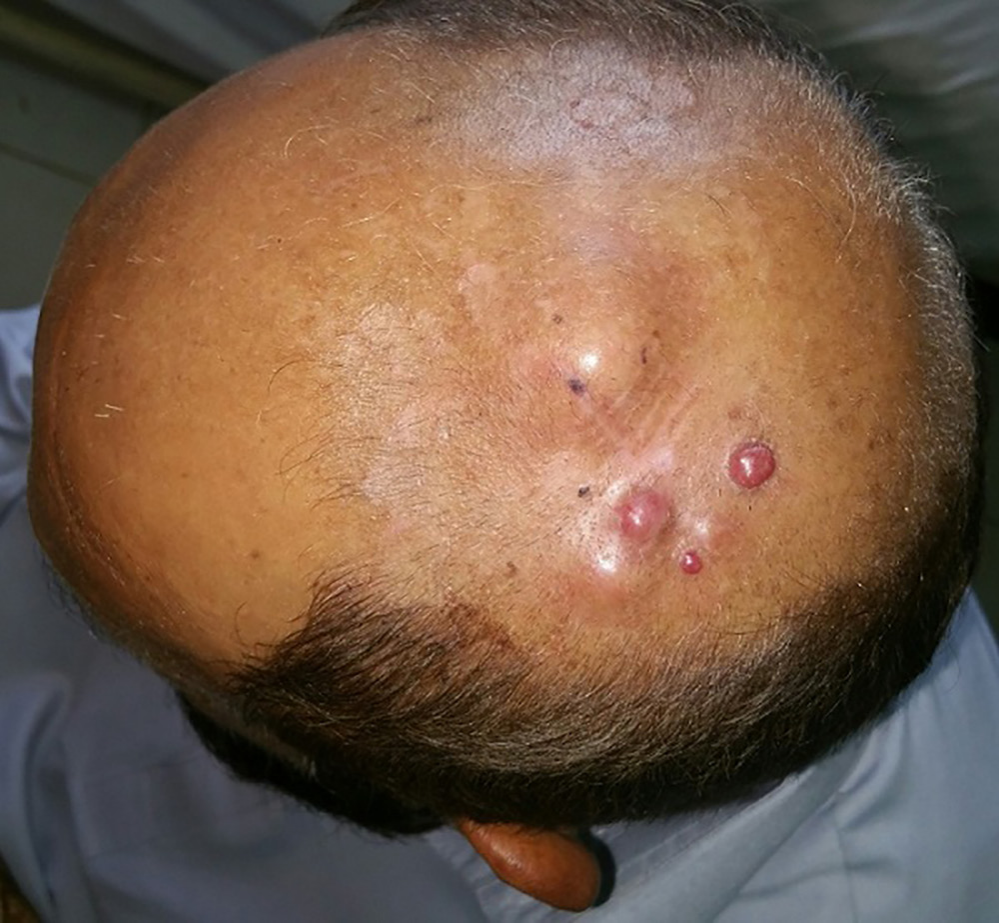
Nodular melanoma affecting the scalp of a 59 years old man

**Fig. 7:**
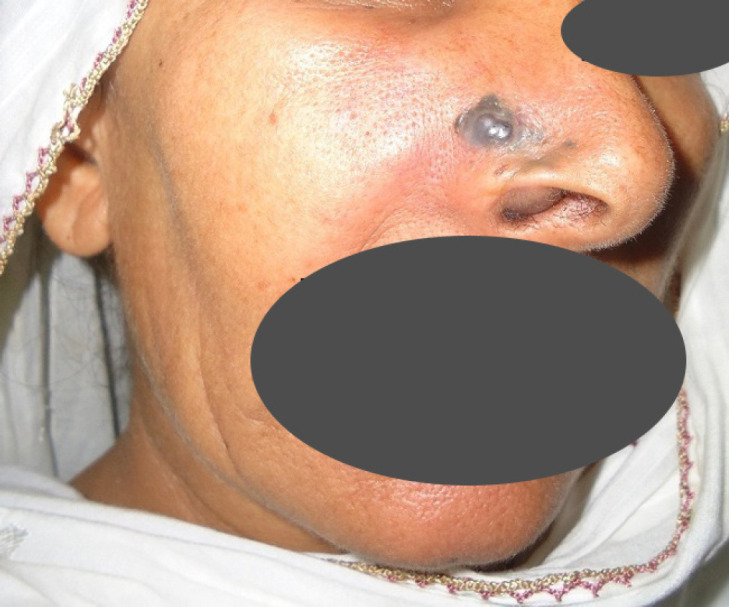
Nodular melanoma involving the right nostril and maxilla in a 57 years old lady

**Fig. 8:**
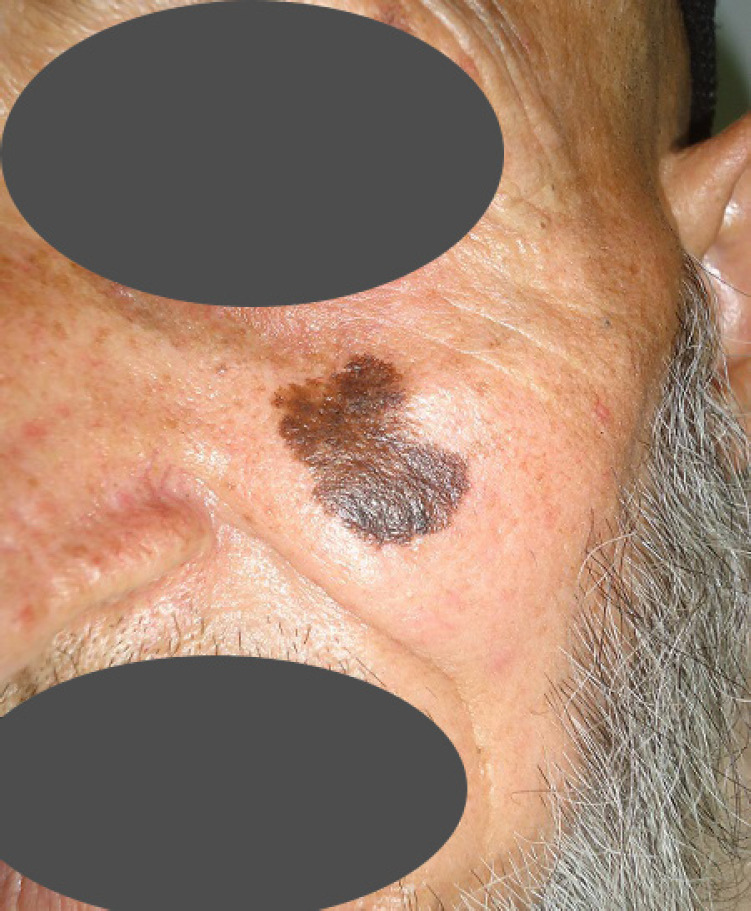
Lentigo maligna melanoma that emerged in a pre-existing lentigo maligna on the left cheek of a 63 years old man

## DISCUSSION

In this study, the overwhelming majority of patients were male. Published literature has variably reported male to female distribution of the patients with CMs. A meta-analysis of 12 studies including 958 Chinese patients reported slight male preponderance; whereas, Yu *et al.* in their series of 82 patients observed a 1:1 gender ratio.^11^ Bray *et al.* from the United States and Canada reported more frequent affliction of males.^4^ Contrary to the finding of more frequent male affliction, several studies have reported more frequent affliction of the females with CMs.^1^^,^^8^^,^^12^

Several studies that reported higher incidence among females have attributed it to their more frequent exposure to environmental risk factors such as the rising trend of more liberal use of tanning beds by women at homes.^7^^,^^13^^,^^14^ In this study, the majority of patients were over 58 years old. Yu *et al.* from China reported median age of the sufferers as 57 years.^11^ de Lima *et al.* from Brazil observed that over 50% of their patients were over 51 years.^12^ Wu *et al.* carried out a detailed comparative analysis of the clinical and demographic features of CMs amongst all major races settled in the United States and found that white and black patients were slightly older (59-63 years) at presentation compared to the Hispanics and other racial groups.^7^

In this study, NM and ALM constituted the bulk of the patients. Different histologic subtypes of CMs have been reported with variable frequencies among different populations across the globe. The majority of studies were carried out on Caucasians observed with SSM as the most frequent histologic type. In glaring contrast to this, studies from most of the South Asia reported ALM as the commonest variety. Wu *et al.* from the US reported SSM as the commonest type among all races except blacks, for whom ALM was the most common.^7^

Similar findings have also been reported by other researchers from the West.^15^^,^^16^ Yu *et al.* from China reported ALM as the commonest (39%) followed by NM (37.8%), LMM (12.2%) and SSM (11.0%).^11^ Other researchers from these countries have also reported ALM as the most common type.^9^^,^^17^^,^^18^ de Lima *et al.* from Brazil reported NM as the commonest type,^12^ whereas Forman *et al.* found that LMM was the most common histologic type accounting for 56% of melanoma cases.^19^

In this study, feet and face represented the most common sites of involvement. The published studies have reported variable frequencies of primary sites of involvement by the CMs. Yu *et al.* in their meta-analysis on Chinese subjects found that foot (52.06%) was the commonest site of affliction, followed by hand (16.89%), head and face (13.45%), limb 13.32% and trunk (11.20%).^11^ de Lima *et al.* from Brazil reported trunk as the most common site of the primary lesion (30.19%), followed by the upper limbs (19.34%), head and neck (18.87%) and lower limbs (13.21%).^12^

Wu *et al.* from the US observed considerable variations among different races living within the US. The trunk remained the site of most frequent involvement among Whites, American Indians and Alaskan natives, whereas the lower limb and hip remained the predominant sites of involvement among blacks and Asians/Pacific islanders. The percentages of the trunk and lower limb/ hip were similar among Hispanics.^7^

In this study, one case had a definite association with preexisting nevus. The nevus-associated melanoma is usually confirmed on histology, where there is a characteristic combination of benign nevus as well as frank melanoma. The nevus-associated melanoma is typically found as SSM and often encountered on the trunk in relatively young individuals. The frequency is variably reported across the published studies, ranging from 4% to 72%.^20^ In this study, the majority of patients presented stage III and IV of CM. 

Several studies from the developed nations showed that majority of their melanoma patients present in early stages. For instance, stage I patients accounted for 82-85%, whereas stage III patients were only 2-5%.^21^^-^^24^ Yu *et al.* in their meta-analysis of Chinese patients found stage I, II, III and IV disease as 17.63%, 43.45%, 26.39% and 10.01%, respectively.^11^ In this study, high mortality was observed as majority of the patients presented with stage III or IV of the disease. Sneyd *et al.* reported CMs occurring in the New Zealanders and Australians over a period of 40 years, spanning from 1968 to 2007. They observed a consistently rising trend in the incidence as well as mortality rates. In the given time period, 6,721 New Zealanders and 29,825 Australians died from melanoma.^3^

Majority of patients in this study presented after 10-weeks of onset of symptoms of the CMs. It is explainable largely on the basis of factors such as lack of awareness about the sinister nature of melanoma, poverty and lack of access to adequate health care facilities. Occurrence of the lesions on less noticeable areas of the body may also contribute to delayed presentation.^25^ Gao *et al.* additionally observed that owing to the low incidence of CMs in Asia and the lack of focus on CMs, many Chinese patients usually presented relatively late to the hospital.^26^

This study reflects a relatively low incidence of CMs in Pakistan. Similar low incidence has been observed among particularly the South Asian populations.^8^^,^^27^ Pigmented skin among the darker races provides protective effects against development of CMs. The dark skin contains a high melanin density which in turn protects from the deleterious effects of UV light emanating from the sun.^7^^,^^28^^-^^30^ In Pakistan, particularly in the rural areas, the male population often wear caps, turbans and head covers, whereas the females mostly wear the traditional parda-attire that keep the sun exposed parts covered. 

This dress code certainly offers protective effects against the damaging effects of the ambient UV light of the sun. CMs more frequently affected males aged ≥58 years. Feet, face, trunk, hands and scalp were the affected anatomical body parts in decreasing order of frequency. NM and ALM were the more common histological subtypes. Majority of the patients presented late with advanced stage disease. 

## CONCLUSION

A future well designed long term prospective study should objectively evaluate the protective effects of these practices against CMs. Awareness about the sinister course of the disease will ensure early presentation and better treatment outcome.

## CONFLICT OF INTEREST

The authors declare no conflict of interest.
